# Natural Disasters: Building a Tsunami Warning System

**DOI:** 10.1289/ehp.113-a90

**Published:** 2005-02

**Authors:** Charles W. Schmidt

The 26 December 2004 tsunami that devastated coastal areas in Indonesia, Malaysia, Thailand, Myanmar, India, the Maldives, Sri Lanka, and Somalia was produced by a magnitude 9.0 earthquake 155 kilometers southwest of northern Sumatra. The tsunami inundated coasts with waves more than 30 feet high every 30–40 minutes for several hours. It killed more than 150,000 people and injured millions, making it one of the worst natural disasters the modern world has ever seen.

Could populations have been warned in advance? Not with the tsunami monitoring networks that exist in the affected area today, say officials with the National Oceanic and Atmospheric Administration (NOAA). Unlike the Pacific Ocean, which is wired for tsunami alerts by the United Nations Intergovernmental Oceanographic Commission, the Indian Ocean is largely devoid of comparable sensor technologies that detect earthquakes and issue tsunami warnings to affected countries. According to NOAA officials, future warnings could be enhanced by the creation of an Indian Ocean tsunami warning center that would deploy coastal tide gauges to measure the amplitude of waves near tsunami source areas such as fault zones and volcanoes, and would establish a communications infrastructure to send and receive alerts.

Tsunami buoys also would be useful in an early warning system, NOAA officials say. At present, a network of six buoys is deployed by NOAA off the Aleutian Islands in the Pacific Ocean. This network, called Deep Assessment and Reporting of Tsunamis, is composed of two parts: a sea floor sensor and a buoy that relays tsunami information to warning centers on the ground by satellite communication. NOAA estimates that about 50 such buoys are needed to adequately cover the world’s oceans. On 6 January 2005 Senator Joe Lieberman (D–CT) proposed a $30 million package to develop these additional buoys, which the U.S. Congress is currently considering. Australian scientists are also designing an Indian Ocean tsunami alert system that is expected to cost US$20 million.

Still more help could come from the sky. On 10 January 2005, NOAA announced that its scientists had measured the height of the December 26 tsunami using data from four Earth-orbiting satellites passing over the affected area at the time. NOAA geophysicist Walter H.F. Smith said in an agency press release that the best application of satellite data to improve tsunami hazard forecasts may be in helping to map the ocean floor from space.

To be truly effective, any tsunami warning system will need to be part of an overall disaster reduction strategy, says Helen Wood, senior advisor for systems and services in NOAA’s National Environmental Satellite, Data, and Information Service. “You don’t want a separate system that segregates tsunamis from earthquakes or cyclones, because populations on the coast are at risk from any and all of those things,” she says.

Wood heads the secretariat for the Global Earth Observation System of Systems, an emerging international coalition that has set disaster reduction as one of nine priority areas [see “Terra Cognita: Using Earth Observing Systems to Understand Our World,” p. A98 this issue]. “We had already identified tsunami and related instrumentation as a necessity,” Wood says. “Now there’s a sense of urgency. [The December 26 tsunami] was a catalyst for action. It’s sad, but disaster often mobilizes people at all levels to take action.”

## Figures and Tables

**Figure:**
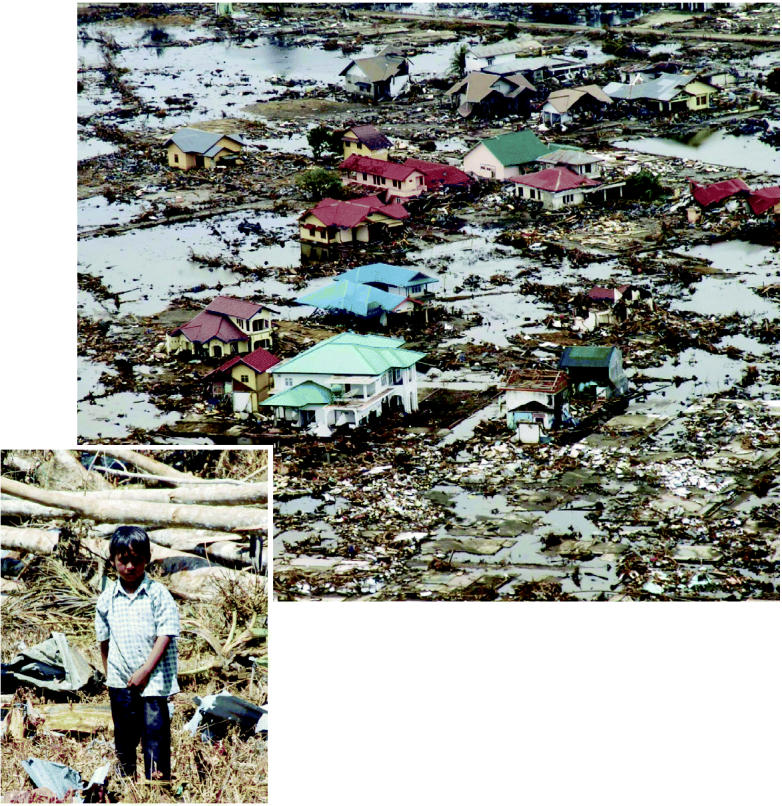
**The tragedy of hindsight.** Similar disasters may be mitigated in the future by better warning sytems.

**Figure:**
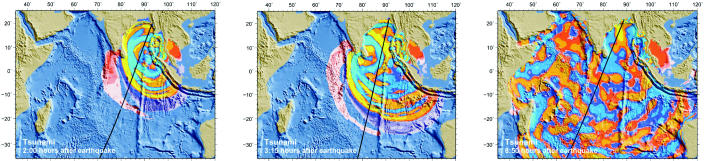
**Waves of destruction.** Composite satellite images created by NOAA measure the tsunami’s height as it spread from the quake epicenter. The ability to make depth surveys from space may lead to improvements in models that forecast the hazardous effects of tsunamis.

